# Estimating gestational age using the anthropometric measurements of newborns in North Shewa Zone public hospitals, Oromia, Ethiopia

**DOI:** 10.3389/fped.2023.1265036

**Published:** 2023-12-06

**Authors:** Ifa Dereje, Mukemil Awol, Asfaw Getaye, Zenebe Tujara, Adugna Alemu, Abdi Negash, Fedasan Alemu, Husen Zakir, Ararsa Dinka, Dejene Edosa, Irean Shigign, Abayneh Tunta, Mathewos Mekonnen, Fikadu Tolesa, Kumera Bekele, Belay Merkeb, Befekadu Oyato, Mekonnin Tesfa

**Affiliations:** ^1^ Department of Medicine, College of Health Sciences, Salale University, Fitche, Oromia, Ethiopia; ^2^ Department of Midwifery, College of Health Science, Salale University, Fitche, Oromia, Ethiopia; ^3^ Department of Nursing, College of Health Science, Salale University, Fitche, Oromia, Ethiopia; ^4^ Department of Medical Laboratory, College of Health Science, Salale University, Fitche, Oromia, Ethiopia; ^5^ Department of Pharmacy, College of Health Science, Salale University, Fitche, Oromia, Ethiopia; ^6^ Department of Public Health, College of Health Science, Salale University, Fitche, Oromia, Ethiopia; ^7^ Department of Biomedical Science, College of Health Science, Woldia University, Woldia, Amhara, Ethiopia

**Keywords:** neonatal anthropometry, North Shewa, gestational age, prematurity, anthropometry

## Abstract

**Background:**

The accurate estimation of gestational age is crucial in identifying prematurity and other health problems in newborns and in providing appropriate perinatal care. Although there are numerous methods for measuring gestational age, they are not always applicable. During these situations, it becomes challenging to ascertain whether a baby has been born prematurely or not. Therefore, this study aims to estimate gestational age by utilizing newborn anthropometric parameters.

**Purpose:**

The objective of this study is to estimate the gestational age of newborns in public hospitals located in the North Shewa Zone of the Oromia Region in Ethiopia, by using anthropometric parameters.

**Methods:**

A cross-sectional study was conducted at a facility from February 2022 to April 2022, using an interview-based questionnaire and anthropometric measurements. The anthropometric parameters that were measured include foot length (FL), mid-upper arm circumference (MUAC), and chest and head circumference (CHC). The study’s sample size had a total of 420 participants. The data were cleaned, edited, manually checked for completeness, and entered into Epi-data version 3.1. Subsequently, the data were transferred into SPSS for analysis. The data were analyzed using descriptive analysis, simple linear regression, and multiple linear regressions. Finally, the data were presented using statements and tables.

**Results:**

There is a significant and positive correlation between anthropometric parameters, including head circumference (*r*: 0.483), MUAC (*r*: 0.481), foot length (*r*: 0.457), and chest circumference (*r*: 0.482) with gestational age. All anthropometric parameters demonstrated positive and significant estimates of gestational age. The combination of the four measurements yielded the strongest estimate of gestational age. Gestational age can be calculated by the formula: Gestational age (Weeks) = 9.78 + 0.209*CHC + 0.607*MUAC + 0.727*FL + 0.322*HC.

**Conclusion:**

Gestational age can be measured using head circumference, mid-upper arm circumference, foot length, and chest circumference. Utilizing the four anthropometric parameters in combination exhibits greater efficacy in estimating gestational age than using them individually. Therefore, it is recommended to use these alternative approaches when standard methods are not applicable.

## 1. Introduction

The average duration of human gestation is 266 days starting, commencing from the day of conception or 280 days from the first day of the last normal menstrual period (LNMP), assuming that a typical menstrual cycle is 28 days and ovulation occurs approximately on day 14 (1). Gestational age (GA) is estimated to determine whether or not a newborn will be born prematurely (2). In the past, GA was predicted by combining historical data from the mother and physical examination (3). Naegele's rule, a simple calculation used to estimate the expected date of delivery, remains the current standard for calculating the length of pregnancy based on the LNMP (4).

The fetal ultrasound scan is considered the gold standard for estimating GA when obtained before 20 weeks of gestation due to its reliance on biometric measurements of the fetus (5). In cases where the gestational age of a newborn has not been determined prior to birth, the New Ballard score (NBS) is commonly employed by health professionals as a means of postnatal prediction of fetal maturation or GA. The NBS criteria depend on physical anatomical changes and neurological criteria, which mainly relies on muscle tone to estimate the GA of the newborn (6, 7).

According to a national study in Ethiopia, only 62% of pregnant women receive at least one antenatal care service, which further reduces the utilization of ultrasound evaluation to determine GA (8). Due to this reason, health professionals mainly rely on LNMP to estimate gestational age. Unfortunately, the use of LNMP is usually unreliable and inaccurate due to various factors such as poor recall, menstrual irregularity, low literacy, and contraceptive usage (9). In addition, the use of estimation of newborn's GA using NBS may be unreliable as its accuracy depends on the skill of the examiner and the condition of the neonate. For example, it is not suitable for use in asphyxiated neonates. It is a complex and subjective assessment that requires the skills of professionals with advanced education (10). Furthermore, the NBS overestimates GA of small for gestational age and preterm neonates (11, 12). This makes it difficult for healthcare providers to make appropriate clinical decisions, such as identifying preterm neonates and post-term and complicated pregnancies (13).

Generally, simple and feasible alternative methods of GA measurements are very important for assessing and managing preterm birth and other neonatal illnesses. Anthropometry can be used for a variety of purposes, such as diagnosing a variety of prenatal and postnatal conditions of the neonate. When scientifically proven to be effective, neonatal anthropometry can be an inexpensive, convenient, easy to measure, and non-invasive tool for assessing the gestational age of newborns. Parameters such as weight, length, and body circumferences are commonly used in clinical practice (14).

There are several proposed studies that found simple and reliable neonatal anthropometric tools in different countries to estimate gestational age. However, according to a multi-centered study conducted by WHO, the mean and 10th percentile for many anthropometric parameters are varied by country and ethnicity (15). This points out that there is a need to find suitable population-specific anthropometric parameters to estimate gestational age.

The presence of an alternative method of postnatal GA assessment of newborns enables healthcare providers to use it when LNMP, US, and NBS are not applicable. Furthermore, due to the simple nature of the measurements, health extension workers in the community and mothers can easily identify GA of the newborns at a household level.

There are few studies conducted on the topic in our country. However, Ethiopia is such a large country with diverse socio-demographic characteristics that a study conducted in one part of the country may not perfectly be applicable to other parts of the country. So, many studies are required in different parts of the country to show how gestational age can be estimated using neonatal anthropometric parameters. Furthermore, this study is the first of its kind in the North Shewa Zone, Oromia Region, Central Ethiopia. Therefore, the purpose of this study is to estimate GA using neonatal anthropometric parameters in public hospitals of the North Shewa Zone, Oromia Region, Ethiopia.

## 2. Material and methods

### 2.1. Study area and study period

The study was conducted in the delivery ward of four public hospitals located in the North Shewa Zone, Oromia Region, Central Ethiopia. The four hospitals include Salale University Comprehensive Specialized Hospital, Kuyu Hospital, Gundo Meskel Hospital, and Sheno Hospital. These hospitals collectively serve approximately 2 million people. The study was conducted from 1 February to 30 April 2022.

### 2.2. Study design and populations

A facility-based cross-sectional design was applied in this study. The source population was all the mothers with their neonates who were delivered at the four hospitals of North Shewa. The selected mothers and their newborn babies delivered in the hospitals and fulfill the eligibility criteria were our study population. Mothers and their neonates delivered in the hospitals with age less than or equal to 24 h were included in the study. The inclusion criteria of the study include: mothers who do not have accurate knowledge of their LNMP, gestational age discrepancy of more than 2 weeks between Naegele's rule and ultrasound scan, women with an irregular menstrual cycle prior to pregnancy, neonates with intra-uterine growth restriction, small for gestational age newborns, twin neonates, newborns with gross congenital anomalies, severe perinatal asphyxia, chronic maternal disease such as hypertension, diabetes mellitus, cardiac disease, and severe anemia, mother positive for TORCH infection, obstetric complications known to compromise fetal growth–eclampsia, and mothers with history of smoking, alcohol consumption, or drug abuse.

### 2.3. Sample size determination and sampling techniques

The sample size was determined using a single population proportion formula, taking the 95% confidence level, 5% margin of error, and 50% (0.50) prevalence (*p*) rate as we could not find a study published on our study topic in a population comparable with our study population during the preparation of our project proposal. After adding 10% of the calculated sample size for non-response rate, the final sample size of the study was 424. The sample size for each hospital was proportionally allocated based on a data on the number of deliveries taken from each hospital. The sample was collected by using consecutive sampling technique.

### 2.4. Data collection methods

The anthropometric parameters were obtained using flexible, non-elastic measuring tape and weighing scale. A physical examination was first conducted on the neonates to assess any visible birth defects which may potentially influence the anthropometric measurements before they were included in the study. The gestational age of the neonate was calculated using “Naegele's formula” (count back 3 months from the first day of LNMP and add 1 year and 7 days).

The mid-upper arm circumference (MUAC), foot length (FL), head circumference (HC), and chest circumference (CHC) were measured according to the standard operating procedures following the good clinical practice guidelines (16). All anthropometric parameters were taken to the nearest 0.1 cm using non-elastic flexible measuring tape. Birth weight was measured with a calibrated digital weighing scale to the nearest 10 g. All measurements were performed three times, and the mean was determined.

### 2.5. Data processing and analysis

The data were cleaned, edited, manually checked for completeness, and entered into Epi-data version 3.1. The data were then transferred into Statistical Package for Social Sciences (SPSS) version 20 for analysis. After categorizing and defining the variables, the descriptive analysis was conducted for each of the independent variable, and the data were presented as figures, frequencies, and percentages. The normality test was conducted to determine the normality of the samples.

The correlation between the neonatal anthropometric parameters and gestational age was measured using the simple linear regression analysis and Pearson's correlation. To identify which combination of variables gives the most accurate predictions of GA, a multiple linear regression analysis was performed using backward elimination method. Linear regression equations were derived as a predictive model for gestational age from neonatal anatomical anthropometric parameters. The multi-collinearity between the independent variables was assessed using the variance inflation factor (VIF). The fitness of regression models was assessed using coefficients of determination (*r*^2^) and residual plots. To examine the predictive accuracy of the regression models, the mean absolute error (MAE) and mean average percentage error (MAPE) were employed. Finally, the data were presented using statements and tables.

### 2.6. Data quality control

To assure the quality of the data, the four data collectors underwent a 2-day training prior to the data collection process regarding the proper way to approach the study subjects, how to use the questionnaire, demonstrate on how to measure neonatal anatomical anthropometric parameters, and regarding ethics during data collection. Anatomical parameters were measured by a flexible non-stretchable tape and recorded to the nearest 0.1 cm. Each measurement was repeated three times in order to maintain reproducibility and reduce measurement errors. A pre-test was performed among 5% of the sample size at Chancho Hospital to assess the integrity of the questionnaire. The quality of the data was maintained by ensuring daily onsite supervision and conducting cross-checking of the collected data during the data collection period.

### 2.7. Ethical considerations

Ethical clearance to conduct the study was obtained from Salale University Institutional review board (IRB). Written letter of permission to data collection was provided to the respective hospital's administrative office and department of obstetrics. The study participants were informed about the purpose and importance of the study, and they were also informed of their right to withdraw at any time during the study period. The mothers of the neonates were interviewed after the mother signed a written informed consent and assent form. The anthropometric measurements do not impose any harm or injury to the newborn or the mother. No personal identifiers, such as name, were collected to maintain the privacy and confidentiality of the participants. To prevent COVID-19, personal protective equipment was worn, and necessary infection prevention techniques were applied during all stages of data collection. This study was conducted in accordance with the Declaration of Helsinki guidelines.

## 3. Results

### 3.1. Demographic characteristics and clinical data of the study participants

A total of 420 mothers and their newborn neonates participated in this study, yielding approximately 99% response rate. Table 1 presents the demographic characteristics and clinical data of the mothers and their newborns at the four included hospitals in North Shewa Zone. The majority of mothers (319/420, 76%) involved in this study are aged between 20 and 34 years. In addition, 326 out of 5,420 (77.6%) mothers belonged to the Oromo ethnic group. Only 121 out of 420 (28.7%) mothers had a college-level of education or higher. In addition, the majority of mothers (265/5,420, 63.1%) were urban residents. Approximately half (212/5,420, 50.7%) of the mothers were housewives. The male to female ratio of newborns was almost equal. In this study, it was found that there were 69/420 (16.4%) preterm newborns using LNMP and 82/420 (19.6%) newborns using ultrasound.

**Table 1:** Demographic characteristics and clinical data of the study participants at four hospitals in North Shewa Zone, Ethiopia (*n* = 420).

Variables	Category	Frequency	Percentage
Age	Below 20	35	8.3%
	20–34	319	76.0%
	35 and above	66	15.7%
Ethnicity	Oromo	326	77.6%
	Amharic	86	20.5%
	Others	8	1.9%
Educational level	No formal education	87	20.8%
	Primary school	136	32.3%
	Secondary school	76	18.2%
	College and above	121	28.7%
Residence	Urban	265	63.1%
	Rural	155	36.9%
Occupation	Unemployed	42	9.8%
	Housewife	212	50.7%
	Gov't employee	97	23.2%
	Private employee	32	7.6%
	Self employed	37	8.8%
Newborn sex	Male	207	49.3%
	Female	213	50.7%
Gestational age using LNMP	Premature (<37 weeks)	69	16.4%
	Mature (≥37 weeks)	351	83.6%
Gestational age using ultrasound	Premature (<37 weeks)	82	19.6%
	Mature (≥37 weeks)	338	80.4%

### 3.2. Gestational age and anthropometric parameters

Table 2 presents information regarding maternal gestational age and neonatal anthropometric parameters in four hospitals in North Shewa Zone. The mean gestational age at the time of birth as measured by LNMP was 38.43(±2.04) weeks. The mean of other anthropometric parameters measured in centimeters is listed as follows: MUAC:10.20 (±0.94) cm, foot length: 7.76(±0.50) cm, head circumference: 31.75 (±1.43) cm, and chest circumference: 31.54 (±1.71) cm. The mean birth weight of the newborns is 3,023.65 (±533.44) g.

**Table 2:** Descriptive statistics of the gestational age of women and anatomical anthropometric parameters of the newborns in four hospitals in North Shewa Zone, Oromia Region, Ethiopia (*n* = 420).

Variables	Minimum	Maximum	Mean	Standard deviation
Gestational age by LNMP (weeks)	29.60	43.40	38.43	2.04
Gestational Age by ultrasound (weeks)	28.70	43.00	38.10	1.96
Mid-upper arm circumference (cm)	7.6	13	10.204	0.94
Foot length (cm)	6.5	9.1	7.76	0.50
Head circumference (cm)	23.9	36.5	31.75	1.43
Chest circumference (cm)	22	36	31.54	1.71
Birth weight (gm)	1,200	4,100	3,023.65	533.44

LNMP, last normal menstrual period; cm, centimeter.

### 3.3. Relationship between anthropometric parameters and gestational age

Table 3 presents the correlation between gestational age and newborn anthropometric parameters in the four hospitals. The head circumference (*r*: 0.483), MUAC (*r*: 0.481), foot length (*r*: 0.457), and chest circumference (*r*: 0.482) are all significantly positively correlated with gestational age.

**Table 3:** Correlation between gestational age and newborn anthropometric parameters in four hospitals in North Shewa Zone, Oromia Region, Ethiopia (*n* = 420).

Parameters	Gestational age by LNMP	
	*r*	*P*
Head circumference	0.483	0.0001
Mid-upper arm circumference	0.481	0.0001
Foot length	0.457	0.0001
Chest circumference	0.482	0.0001

*r*, Pearson's correlation coefficient; *P*, level of significance (<0.05).

### 3.4. Estimation of gestational age using anthropometric parameters

An estimation of gestational age using linear regression was performed. All anthropometric parameters used in this study were found to be positive and significant estimates of gestational age. Table 4 shows the estimation of gestational age using newborn anthropometric parameters. Regression equation to calculate sample size using each anthropometric parameter is presented in the table below. With regard to how the head circumference estimates for gestational age, a 1 cm increase in head circumference increases gestational age by 0.689 week after adding 16.55 weeks. In addition, a 1 cm increase in MUAC increases, gestational age by 1.04 week with a constant of 27.78 weeks. After taking or adding 20.32 weeks of gestational age, each 1 cm increase in chest circumference increases gestational age by 0.574 week. This study also revealed the combination of the four anthropometric parameters to calculate gestational age. Gestational age can be calculated using the following formula: gestational age (GA) (in weeks) 9.78 + 0.209*CHC + 0.607*MUAC + 0.727*FL(cm) + 0.322*HC.

**Table 4:** Estimation of gestational age using newborn anthropometric parameters in four hospitals of North Shewa Zone, Oromia Region, Ethiopia (*n* = 420).

Parameters	*r*	*r* ^2^	Adjusted *r*^2^	SEE	Regression equations GA (weeks)	Sig.
Head circumference	0.483	0.233	0.232	1.79	16.55 + [0.689*HC(cm)]	0.0001
Mid-upper arm circumference	0.481	0.231	0.230	1.79	27.78 + [1.04*MUAC(cm)]	0.0001
Foot Length	0.457	0.209	0.207	1.82	23.84 + [1.88*FL(cm)]	0.0001
Chest circumference	0.482	0.232	0.230	1.79	20.32 + [0.574*CHC(cm)]	0.0001
HC,MUAC,FL and CHC	0.639	0.409	0.404	1.58	9.78 + [0.209*CHC(cm)] + [0.607*MUAC(cm)] + 0.727*FL(cm) + [0.322*HC(cm)]	0.0001

*r*, Pearson's correlation coefficient; HC, head circumference; cm, centimeter; GA, gestational age; MUAC, mid-upper arm circumference; FL, foot length; CHC, chest circumference; MAE, mean absolute error; Sig, level of significance (*P* < 0.05).

### 3.5. Measurement of the predictive capacity of the formulated regression models

Table 5 presents the predictive accuracy measures of the formulated regression models using the mean absolute error (MAE) and mean average percentage error (MAPE). This study revealed that both MAE and MAPE show that all of the formulated regression models had the capacity to estimate the gestational age. According to the MAE results, using the combination of all of the four parameters demonstrated the best predictive accuracy (MAE = 1.21) followed by CHC (MAE = 1.35). The predictive accuracy of the model using MAPE indicated that the model using all of the four parameters had the lowest error (MAPE = 3.15%), followed by the model of CHC (MAPE = 3.55).

**Table 5:** Predictive accuracy measure of the regression models for estimation of gestational age using newborn anthropometric parameters in four hospitals of North Shewa Zone, Oromia Region, Ethiopia.

No.	Models	MAE	MAPE	Sig.
1.	16.55 + [0.689 × HC(cm)]	1.38	3.62	0.0001
2.	27.78 + [1.04 × MUAC(cm)]	1.38	3.62	0.0001
3.	23.84 + [1.88 × FL(cm)]	1.39	3.65	0.0001
4.	20.32 + [0.574 × CHC(cm)]	1.35	3.55	0.0001
5.	9.78 + [0.209 × CHC(cm)] + [0.607 × MUAC(cm)] + [0.727 × FL (cm)] + [0.322 × HC (cm)]	1.21	3.15	0.0001

HC, head circumference; cm, centimeter; GA, gestational age; MUAC, mid-upper arm circumference; FL, foot length; CHC, chest circumference; MAE, mean absolute error; Sig, level of significance (*P* < 0.05).

## Discussion

This study aimed to estimate gestational age using newborn anthropometric measurements including HC, CHC, FL, and MUAC in North Shewa Zone public hospitals in the Oromia Region of Central Ethiopia. The study was conducted in the Obstetrics Department within 24 h after delivery.

Based on the findings of this study, the mean and standard deviation of MUAC was 10.20 (±0.94) cm. This finding (MUAC) is consistent with the findings from a study conducted in Jimma University Medical Center which found the mean and standard deviation of MUAC as 10.4 (±1.0) cm (17). However, it is different from the findings of a study conducted in Vietnam which reported a mean and standard deviation of 8.9 (±1.1) cm (18). The possible reason for this discrepancy may be due to difference in socio-demographic characteristics and population variation between these studies. The above finding is also different from the findings of a study conducted in Eastern Ethiopia, which reported a mean and standard deviation of 8.7 (±1.4) cm (19). The possible reason for this difference may be due to the difference in socio-demographic characteristics between the two studies.

The mean and standard deviation of FL found in this study was 7.76 (±0.50) cm. This finding is similar with the findings from a study conducted in Vietnam which found a mean and standard deviation of 7.4 (±0.6) cm (18), Jimma University Medical Center 7.8 (±0.5) cm, Gondar 7.41 (±0.68) cm (17) and Dire Dawa 7.84 (±1.0) cm (19).

The mean and standard deviation of head circumference reported in our study was 31.75 (±1.43) cm. The above finding is different from the findings of a study conducted in Dire Dawa which reported 33.4 (±2.2) cm. This variation may be due to the difference in the socio-demographics of the two studies. This finding was also different from the findings of a study conducted in Brazil which reported a mean and standard deviation of head circumference of 34.80 (±1.35) cm for male newborns and 34.18 (±0.89) cm for females (20). This discrepancy may be due to the difference in the study population. Our study included both term and preterm newborns, while the study conducted in Brazil involved only term newborns. Our finding is also inconsistent with the findings of a study conducted in India which reported a mean and standard deviation of 33.64 and 1.40 cm, respectively (21).

Our study revealed a chest circumference mean and standard deviation of 31.54 (±1.71) cm. This finding is consistent with the findings of a study conducted in Dire Dawa (Ethiopia) and India, which reported 31.7 (±3.09) cm and 31.85 cm (±2.19) cm, respectively (19, 21). This finding is close to the findings of a study conducted in Jimma University Medical Center, which showed a mean and standard deviation of 32.7 (±2.3) cm (17). However, our finding is inconsistent with the findings of a study conducted in Brazil, which reported a mean and standard deviation of 34.13 (±1.46) cm for boys and 33.51 (±1.39) cm for girls (20). The possible reason for this discrepancy is the difference in the study population. The study population in the study conducted in Brazil included only term newborns, while our study included both preterm and term newborns.

In this study, the four anthropometric parameters, HC (*r* = 0.483), CHC (*r* = 0.482), FL (*r* = 0.457), and MUAC (*r* = 0.481), had a positive significant correlation with gestational age. This finding is consistent with the findings of the studies conducted in India (10), Dire Dewa Administration in Eastern Ethiopia (19), Dessie Referral Hospital in Ethiopia (2), Belgium (22, 23), and Gondar in Ethiopia (24).

In this study, the head circumference was significantly and positively associated with gestational age (*r* = 0.483). This finding is consistent with the findings of the studies conducted in China (25), three studies in India (*r* = 0.52) (10, 21, 26), Dessie in Ethiopia (*r* = 0.149) (2), and Dire Dawa in Ethiopia (19). Similarly, the chest circumference was significantly positively associated with gestational age (*r* = 0.482). This result is supported by the findings from a study conducted in Dessie in Ethiopia (*r* = 0.143) (2) and Dire Dawa in Ethiopia (*r* = 0.39) (19). A study conducted in India also found significant positive correlation between CHC and GA (*r* = 0.763) (21).

This study revealed that foot length and gestational age were significantly positively correlated with each other (*r* = 0.457). This finding is supported by the studies conducted in Dire Dawa in Ethiopia (*r* = 0.48) (19), Belgium (22, 23), Gondar (Ethiopia) (24, 27) and India (*r* = 0.43) (10). According to the results of our study, MUAC was positively and significantly associated with gestational age (*r* = 0.481). This finding is in line with the findings of the studies conducted in Dessie in Ethiopia (2), Dire Dawa in Ethiopia (*r* = 0.35) (19), and India (*r* = 0.64) (10).

Concerning the strength of association in the present study, HC (*r* = 0.483) exhibited a relatively strong correlation with gestational age followed by CHC (*r* = 0.482). This finding was consistent with the findings of a study conducted by Das et al. (26), which revealed that head circumference had the strongest association (*r* = 0.863) with gestation age followed by CHC (0.859). However, this study is inconsistent with the study conducted in Dessie Ethiopia (2), Dire Dawa Ethiopia (19), and Yadav et al. (28), which found MUAC and foot length to exhibit the highest correlation with gestational age, respectively. This discrepancy may be due to the demographic profile and sample size differences.

A regression model was created to estimate gestational age using anthropometric parameters. Regression equation to estimate gestational age was 16.55 + 0.689*HC (cm) for HC, 27.78 + 1.04*MUAC (cm) for MUAC, 23.84 + 1.88*FL (cm) for FL, and 20.32 + 0.574*HC (cm) for CHC. An equation with the strongest association with the lowest standard error of estimate (1.58) and highest correlation coefficient (0.639) was obtained by using the combination of the four anthropometric measurements. The equation is formulated as GA in weeks = 9.78 + 0.209*CHC (cm) + 0.607*MUAC (cm) + 0.727*FL (cm) + 0.322*HC (cm). Three anthropometric parameters (HC, MUAC, and CHC) had almost equal strength of association while foot length had the least strength of association. This finding was in line with the findings of a study conducted in Dessie, Ethiopia (2). This shows that using the combination of many anthropometric parameters would lead to a better prediction of gestational age than individual anthropometric measurements.

The measure of predictive accuracy of all regression models in the present study was studied. The findings of our study revealed the lowest MAE (1.21) and MAPE (3.15) from the model containing all anthropometric parameters. This finding is consistent with the findings of a study conducted in Dessie Referral Hospital (2). This indicates that using the combination of parameters is better than using a single parameter.

### Strength and limitations of the study

The study has certain strengths and limitations. The findings of this study will improve the identification of gestational age in developing countries including Ethiopia. The formulated equations are simple, quick, and cost-effective and could be used by healthcare workers at the community level to identify preterm newborns and then refer them to higher healthcare institutions for further management. The measurements were performed by trained healthcare professionals, but the tool that will be used by healthcare workers at the community level and their skills may vary. Our equation using multiple variables may lead to some errors in practical use, and training may be needed to reduce error. In addition, our tool may not be suitable for cases demanding precise identification of small differences in gestational age.

## Conclusion and recommendations

Based on the results of this study, all the four anthropometric parameters (HC, CHC, MUAC, and FL) showed a significant and positive relationship with gestational age. These anthropometric parameters can be used individually or together to determine gestational age. The regression equation GA: [0.209*CHC (cm)] + [0.607*MUAC (cm)] + 0.727*FL (cm) + [0.322*HC (cm)] can be used to estimate gestational age when the gestational age of the mother cannot be determined using the common methods. Therefore, based on the findings of our study, we recommend the healthcare providers to consider using HC, MUAC, FL, and CHC when standard methods are not applicable. In addition, we recommend conducting large-scale studies with larger sample size and different study designs across various regions within the country to implement this gestational age method at the national level.

## Data Availability

The raw data supporting the conclusions of this article will be made available by the authors, without undue reservation.
